# A phase I dose-escalation trial of stereotactic body radiotherapy using 4 fractions for patients with localized prostate cancer

**DOI:** 10.1186/s13014-019-1369-y

**Published:** 2019-09-02

**Authors:** Takuro Kainuma, Shogo Kawakami, Hideyasu Tsumura, Takefumi Satoh, Ken-ichi Tabata, Masatsugu Iwamura, Kazushige Hayakawa, Hiromichi Ishiyama

**Affiliations:** 1 Department of Radiation Oncology, Kitasato University School of Medicine, 1-15-1 Kitasato, Sagamihara, Kanagawa Japan; 2 Department of Urology, Kitasato University School of Medicine, 1-15-1 Kitasato, Sagamihara, Kanagawa Japan

**Keywords:** Prostate cancer, Stereotactic body radiotherapy, Dose-escalation

## Abstract

**Purpose:**

To report results from our phase I dose-escalation study of stereotactic body radiotherapy (SBRT) using 4 fractions for patients with localized prostate cancer.

**Materials & methods:**

Fraction sizes of 8 Gy, 8.5 Gy, and 9 Gy were defined as levels 1, 2, and 3. The prescribed dose was delivered to at least 95% of the planning target volume. Image-guided, intensity-modulated radiotherapy was delivered to all patients. Dose-limiting toxicity (DLT) was defined as acute toxicity of Grade 3 or higher. The maximum tolerated dose (MTD) was defined as the level at which ≥30% of patients showed DLT. The recommended dose (RD) was defined to be one dose level below the MTD. If no patients at level 3 showed DLT, level 3 was defined as the recommended dose (RD).

**Results:**

Nine patients were enrolled in each level. All patients were low or intermediate risk. Median durations of follow-up for patients at levels 1–3 were 48.9 months, 42.6 months, and 18.4 months, respectively. Protocol treatment was completed for all patients. No patient showed DLT at each dose level. Level 3 was therefore designated as the RD for the phase II study. Although most toxicities were Grade 1, genitourinary toxicity was common compared to gastrointestinal toxicity. Three-year biochemical control rate was 90.3%.

**Conclusion:**

The dose level of 36 Gy in 4 fractions with a 2-day break was tolerable and highly encouraging for SBRT of localized prostate cancer. The phase II trial to confirm the efficacy and toxicity of this treatment is now on going.

**Trial registration:**

UMIN, UMIN000010236. Registered 13 March 2013.

## Background

The rapid advent of stereotactic body radiotherapy (SBRT) has changed our practice for treating localized prostate cancer. Most patients choose SBRT when informed about the two options of conventional intensity-modulated radiotherapy (IMRT) or SBRT, based not only on the short treatment time, but also the encouraging results.

Although the majority of reported series have used 35–37 Gy in 5 fractions [1–3], the optimal size and number of fractions have not yet been established for SBRT of the prostate. We started this dose-escalation trial in 2011 referring to high-dose rate brachytherapy (HDR) series using 4 fractions that had already described long-term results [4–6]. Compared to 5 fractions, we believe that our 4-fraction schedule offers several benefits.

In this study, we report results from our Phase I dose-escalation study of SBRT using 4 fractions for patients with localized prostate cancer.

## Materials & methods

Eligible patients had to have histologically confirmed adenocarcinoma of the prostate with Gleason score ≤ 7, initial PSA ≤10 ng/ml, and clinical T1-T2b with neither lymph node nor distant metastases according to the UICC TNM classification version 7. Eligibility also required that patients be ≥20 years old with Eastern Cooperative Oncology Group performance status 0–1, white-cell count ≥4,000/mm^3^, hemoglobin concentration ≥ 10.0 mg/dL, and platelet count ≥100,000/mm^3^. Patients were excluded from this study if they met any of the following criteria: 1) history of radiotherapy, chemotherapy, or hormonal therapy; 2) deteriorated organ functions; 3) active malignancy at another site; 4) poorly controlled diabetes mellitus; 5) acute inflammatory disease; 6) cerebral stroke diagnosed with 6 months; 7) psychiatric disorder; or 8) continuous administration of steroidal drugs. Pretreatment evaluations included chest radiography, computed tomography (CT) of the abdomen and pelvis, and magnetic resonance imaging of pelvis.

Table 1 shows the dose-escalation schedule. Nine patients at each level were assigned to receive the SBRT in escalating doses. Image-guided, intensity-modulated radiotherapy using conventional linac or tomotherapy was delivered with a 2-day break (Saturday and Sunday). All patients were implanted with fiducial markers at the apex and base of the prostate before CT simulation. Clinical target volume (CTV) covered the prostate gland and proximal 1 cm of seminal vesicles. Planning target volume (PTV) was defined as the CTV plus 5-mm margins except posteriorly (3-mm). Prescribed dose was delivered to at least 95% of the PTV. Outer circumference of the rectum was delineated from the recto-sigmoid junction to the caudal edge of the ischium or 3 cm above the anal verge, whichever was lower. Outer circumferences of the bladder, femoral head, and small intestine (if it was close to the PTV), were also delineated. Dose-volume constraints for normal tissues were calculated from guidelines for conventional fractionation experiences [7, 8] (Table 2).

**Table 1:** Dose-escalation schedule

	Fraction size (Gy)	Number of fractions	Total dose (Gy)
Level 1	8.0	4	32
Level 2	8.5	4	34
Level 3	9.0	4	36

**Table 2:** Dose-volume constraints for normal tissues

Normal tissue dose-volume	Constraint
Rectum	
V31 Gy	25%
V28 Gy	40%
V24 Gy	55%
V20 Gy	65%
Bladder	
V28 Gy	30%
V24 Gy	50%
Femoral head	
Maximum	28 Gy
Small intestine	
Maximum	24 Gy

*V* volume

Dose-limiting toxicity (DLT) was defined as acute toxicity of at least Grade 3. The maximal tolerated dose was defined as the level at which 30% or more of patients showed DLT. If one or two of the 9 patients had DLT, the dose was escalated to the next level. If three or more patients had DLT, the dose was defined as the maximum tolerated dose (MTD). The recommended dose (RD) was defined to be one dose level below the MTD. However, if no patients at level 3 showed DLT, the level 3 was defined as the RD.

Adverse events were evaluated according to the National Cancer Institute’s Common Terminology Criteria for Adverse Events (NCI-CTCAE) version 4.0 and Radiation Therapy Oncology Group scale [9]. In addition, the Expanded Prostate Cancer Index Composite (EPIC) [10] was used for assessment of health-related quality of life (QOL). Follow-up evaluations were performed at 1, 3, 6, 9, and 12 months until 1 year after treatment, and at 6-month intervals thereafter.

## Results

Nine patients were enrolled in each level. Patient characteristics are shown in Table 3. All patients were low or intermediate risk. One-third of patients received hormonal therapy for 6–22 months (median, 8.5 months). One patient with T2c and 5 patients with initial PSA > 10 ng/ml (range, 12.1–17.5 ng/ml) were included in the analysis after confirmation of acceptable minor violations. Two patients had histories of abdominal surgery (sigmoid colon cancer and gastric cancer). One patient had a history of transurethral resection of the bladder tumor. One patient had a history of holmium laser nucleation of the prostate. Median follow-up for patients at levels 1–3 was at 48.9 months, 42.6 months, and 18.4 months, respectively.

**Table 3:** Patient characteristics

Variables	Values	SD
Age (y.o.)	71.9	4.74
iPSA (ng/mL)	7.94	3.66
Gleason score		
3 + 3	11	
3 + 4	6	
4 + 3	10	
T stage		
1c	12	
2a	11	
2b	3	
2c	1	
Positive cores (%)	32.2	29.3
Hormonal therapy		
Yes	10	
No	17	
CTV (cm^3^)	47.7	22.7
PTV (cm^3^)	96.9	35.4

*Abbreviations*: *iPSA* initial prostate specific antigen, *SD* Standard deviation, *CTV* Clinical target volume, *PTV* Planning target volume, Values are mean or number

Protocol treatment was completed for all patients. No patient experienced DLT at any dose level. As a result, level 3 was designated as the RD for the phase II study. Acute and late toxicities at each level are shown in Tables 4 and 5. Although most toxicities were Grade 1, genitourinary toxicity was common compared to gastrointestinal toxicity. One patient at level 2 had a 1-week hospital stay because of prostatitis after implantation of fiducial markers.

**Table 4:** Acute toxicities

	Level 1 (*n* = 9)				Level 2 (*n* = 9)				Level 3 (*n* = 9)			
	G1		G2		G1		G2		G1		G2	
RTOG												
GU	3	(33.3%)	3	(33.3%)	4	(44.4%)	1	(11.1%)	7	(77.8%)	0	(0.0%)
GI	3	(33.3%)	0	(0.0%)	6	(66.7%)	0	(0.0%)	3	(33.3%)	1	(11.1%)
Micturition pain	1	(11.1%)	0	(0.0%)	3	(33.3%)	0	(0.0%)	1	(11.1%)	0	(0.0%)
Frequency	2	(22.2%)	3	(33.3%)	4	(44.4%)	1	(11.1%)	6	(66.7%)	0	(0.0%)
Urinary incontinence	1	(11.1%)	0	(0.0%)	0	(0.0%)	0	(0.0%)	0	(0.0%)	0	(0.0%)
Retention	1	(11.1%)	1	(11.1%)	2	(22.2%)	0	(0.0%)	1	(11.1%)	0	(0.0%)
Hematuria	0	(0.0%)	0	(0.0%)	0	(0.0%)	0	(0.0%)	0	(0.0%)	0	(0.0%)
Stricture	0	(0.0%)	0	(0.0%)	0	(0.0%)	0	(0.0%)	0	(0.0%)	0	(0.0%)
Proctitis	2	(22.2%)	0	(0.0%)	3	(33.3%)	0	(0.0%)	3	(33.3%)	0	(0.0%)
Fecal incontinence	0	(0.0%)	0	(0.0%)	0	(0.0%)	0	(0.0%)	0	(0.0%)	0	(0.0%)
Diarrhea	1	(11.1%)	0	(0.0%)	3	(33.3%)	0	(0.0%)	0	(0.0%)	0	(0.0%)
Rectal hemorrhage	1	(11.1%)	0	(0.0%)	4	(44.4%)	0	(0.0%)	1	(11.1%)	1	(11.1%)

*GU* Genitourinary toxicity, *GI* Gastrointestinal toxicity, *RTOG* Radiation therapy oncology group

**Table 5:** Late toxicities

	Level 1 (*n* = 9)				Level 2 (*n* = 9)				Level 3 (*n* = 9)			
	G1		G2		G1		G2		G1		G2	
RTOG												
GU	3	(33.3%)	1	(11.1%)	4	(44.4%)	0	(0.0%)	4	(44.4%)	0	(0.0%)
GI	2	(22.2%)	0	(0.0%)	0	(0.0%)	0	(0.0%)	2	(22.2%)	1	(11.1%)
Micturition pain	0	(0.0%)	0	(0.0%)	1	(11.1%)	0	(0.0%)	0	(0.0%)	0	(0.0%)
Frequency	4	(44.4%)	0	(0.0%)	4	(44.4%)	0	(0.0%)	4	(44.4%)	0	(0.0%)
Urinary incontinence	1	(11.1%)	0	(0.0%)	1	(11.1%)	0	(0.0%)	0	(0.0%)	0	(0.0%)
Retention	0	(0.0%)	1	(11.1%)	0	(0.0%)	0	(0.0%)	0	(0.0%)	0	(0.0%)
Hematuria	0	(0.0%)	0	(0.0%)	1	(11.1%)	0	(0.0%)	0	(0.0%)	0	(0.0%)
Stricture	0	(0.0%)	0	(0.0%)	0	(0.0%)	0	(0.0%)	0	(0.0%)	0	(0.0%)
Proctitis	0	(0.0%)	0	(0.0%)	0	(0.0%)	0	(0.0%)	1	(11.1%)	0	(0.0%)
Fecal incontinence	0	(0.0%)	0	(0.0%)	0	(0.0%)	0	(0.0%)	0	(0.0%)	0	(0.0%)
Diarrhea	0	(0.0%)	0	(0.0%)	0	(0.0%)	0	(0.0%)	0	(0.0%)	0	(0.0%)
Rectal hemorrhage	2	(22.2%)	0	(0.0%)	0	(0.0%)	0	(0.0%)	1	(11.1%)	1	(11.1%)

*GU* Genitourinary toxicity, *GI* Gastrointestinal toxicity, *RTOG* Radiation therapy oncology group

Three patients experienced PSA recurrence 17 months, 24 months, and 57 months after treatments. The 3-year biochemical control rate was 90.3%. One patient at level 2 showed local recurrence diagnosed by biopsy 3 years after treatment. Among the three patients with PSA recurrence, only the patient who had local recurrence received salvage hormonal therapy. Two patents died, due to pancreatic cancer and suspected duodenum tumor.

**Fig. 1:**
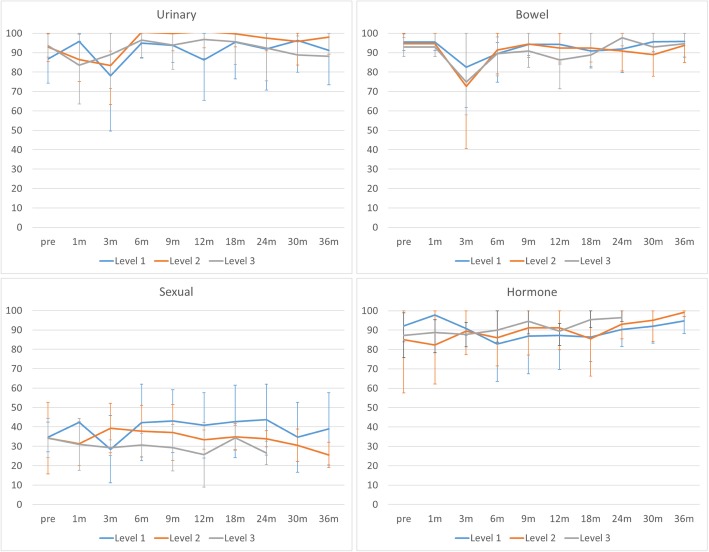
Patient-reported outcomes assessed by Expanded Prostate Cancer Index Composite. A temporary drop and subsequent recovery are seen within the first 3 months after treatment regarding urinary and bowel functions. No significant differences in scores are seen among the 3 dose levels. Error bars represent standard deviations

Figure 1 shows patient-reported outcomes assessed by EPIC. Regarding urinary and bowel function, drops and subsequent recovery were seen within the first 3 months after treatment. No significant difference in EPIC score was detected among the 3 dose levels.

## Discussion

The 2019 guideline [11] from American Society for Radiation Oncology, the American Society for Clinical Oncology, and the American Urological Association “conditionally” recommends ultra-hypofractionated radiotherapy using 5Gy or more per fraction. However, recently published randomized control trial [12] would change the situation. Widmark et al. compared a conventional fractionation schedule (78Gy in 39 fractions) with an ultra-hypofractionation schedule (42.7Gy in 7 fractions) with 1200 prostate cancer patients. There was no differences between the two-schedules regarding tumor control and late toxicity. Non-inferiority of ultra-hypofractionation to conventional fractionation was clearly demonstrated by this trial. Therefore, SBRT using >5Gy per fraction would become more common in the near future.

Table 6 shows reported dose-escalation trials of SBRT for localized prostate cancer [13, 14, 15]. Because all those trials used 5 fractions [1–3], it is unsurprising that the majority of subsequent trials and current clinical practice have also used 5 fractions. We believe, however, that our 4-fraction schedule offers several benefits compared to a 5-fraction schedule.

**Table 6:** Dose escalation trials for stereotactic body radiotherapy for prostate cancer patients

Author	Year	n	Dose and fractionation	Follow-up	Biochemical control	Acute GU	Acute GI	Lage GU	Late GI
Zelefsky	2019	136	32.5Gy - 40Gy in 5fx	3.5–5.9 years	5 years: 85–100%	G2: 8.3–22.9% ≥G3: 0%	G2: 0–11.4% ≥G3: 0%	G2: 23.3–31.4% ≥G3: 0–2.9%	G2: 0% ≥G3: 0%
Hannan	2016	91	45Gy–50Gy in 5fx	66–74 months	5 years: 90.9–100%	G2: 6.7–33.3% ≥G3: 0%	G2: 6.7–26.7% ≥G3: 0–3.2%	G2: 19.7–26.7% ≥G3: 0–6.7%	G2: 0–18% ≥G3: 0–9.9%
McBride	2012	45	36.25Gy–37.5Gy in 5fx	44.5 months	3 years: 100%	G2: 19% ≥G3: 0%	G2: 7% ≥G3: 0%	G2: 17% ≥G3: 2%	G2: 7% ≥G3: 5%
Present study		27	32Gy–36Gy in 4fx	42 months	3 years: 90.3%	G2: 0–33% ≥G3: 0%	G2: 0–11% ≥G3: 0%	G2: 0–11% ≥G3: 0%	G2: 0–11% ≥G3: 0%

*Abbreviations*: *GU* Genitourinary toxicity, *GI* Gastrointestinal toxicity

First, additional tumor control effects might be obtained for the same level of toxicity. Regarding late rectal toxicity, on an assumption [16] of α/β = 5, the schedule of 39.25 Gy in 5 fractions equals our 36 Gy in 4 fractions (equivalent dose in 2-Gy fractions: EQD = 72 Gy). However, regarding prostate cancer, and on the assumption [17] of α/β = 1.5, the biological effect of 39.25 Gy in 5 fractions (EQD = 104.8 Gy) was lower than with our 36 Gy in 4 fractions (EQD = 108 Gy). The 4-fraction schedule thus provided a 3-Gy benefit for tumor control at the same level of rectal toxicity.

Second, a 4-fraction schedule showed no treatment carry-over from the previous week. For example, the number of operating weeks is 50 in the Japanese calendar for 2019. However, 11 of these 50 weeks (22%) have only 4 operating days because of national holidays. As a result, one-fifth of patients would be carried over to the next week if a 5-fraction schedule was applied. Such carry-over increases work load and might lead to treatment errors. Our 4-fraction schedule could resolve this problem.

Third, although a difference of one fraction might be small for a single patient, the difference in total cost would not be negligible for high-volume centers such as academic institutes.

Regarding toxicity, we believe that a 2-day break has some mitigating effects. King et al. reported a significantly lower toxicity rate with a schedule of three times a week compared to a consecutive daily schedule [18]. We therefore inserted a 2-day break among the 4-fraction schedule. As our study showed, the 4-fraction schedule with a 2-day break was acceptable regarding acute and late toxicities. In addition, health-related QOL as assessed by EPIC was also acceptable under this schedule.

## Conclusions

The dose level of 36 Gy in 4 fractions with a 2-day break was tolerable and highly encouraging for SBRT of localized prostate cancer. The phase II trial to confirm the efficacy and toxicity of this treatment is now on going.

## Data Availability

Data sharing not applicable to this article as no datasets were generated or analyzed during the current study.

## Abbreviations

CT: Computed tomography
CTV: Clinical target volume
DLT: Dose-limiting toxicity
EPIC: Expanded Prostate Cancer Index Composite
HDR: High-dose rate brachytherapy
IMRT: Intensity-modulated radiotherapy
MTD: Maximum tolerated dose
NCI-CTCAE: National Cancer Institute’s Common Terminology Criteria for Adverse Events
PTV: Planning target volume
QOL: Quality of life
RD: Recommended dose
SBRT: Stereotactic body radiotherapy
